# Macrophages on the wrinkle: Exploring microscale interactions with substrate topography

**DOI:** 10.1063/5.0215563

**Published:** 2024-06-11

**Authors:** Francesca Cecilia Lauta, Luca Pellegrino, Roberto Rusconi

**Affiliations:** 1 Department of Biomedical Sciences, Humanitas University, 20072 Pieve Emanuele, Italy; 2 IRCCS Humanitas Research Hospital, 20089 Rozzano, Italy

## Abstract

Macrophages play pivotal roles in the immune response, participating in both inflammatory and pro-healing processes. Like other cells, macrophages continually survey their microenvironment through mechanosensing, adapting their intracellular organization in response to mechanical signals. In this study, we elucidate how macrophages perceive the topographical cues of wrinkled surfaces through actin-based structures, which align with the main pattern direction, thus modulating cell cytoskeletal dynamics. Given that such alterations may regulate mechanosensitive gene expression programs, exploring cellular responses to biomaterial design becomes crucial for developing biomaterials that mitigate adverse reactions.

## I. METHODS

Polydimethylsiloxane (PDMS) is a versatile elastomeric polymer extensively utilized in biomedicine and microfluidics due to its outstanding optical clarity, resistance to degradation, biocompatibility, as well as chemical and thermal stability.^1^ By adjusting the ratio between the elastomer base (silicon) and the curing agent (cross-linker), substrates with elastic moduli ranging from a few kPa to tens of MPa can be obtained,^2^ covering a range suitable for mimicking both human tissues and medical implants, such as breast prostheses and polymeric heart valves.^3–5^ Higher elastic moduli can be achieved through plasma oxidation of PDMS samples. Additionally, PDMS can be engineered to create surfaces with various textures, including micro-scale sinusoidal patterns, which spontaneously arise through mechanical buckling deformation and plasma oxidation techniques.^6^

**FIG. 1.**
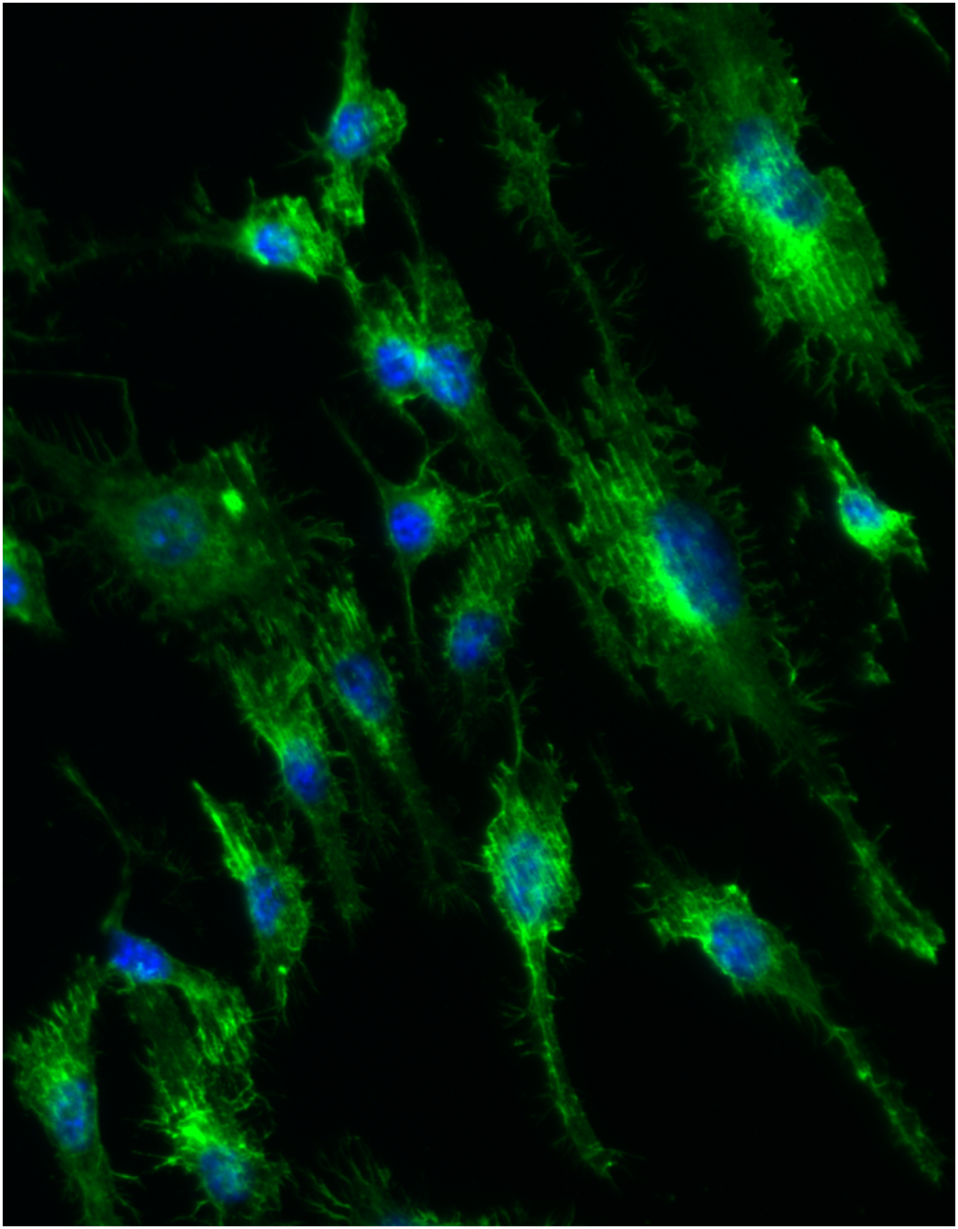
Visualization of macrophage response to wrinkled substrates. To investigate the influence of wrinkled substrates on macrophage cytoskeleton dynamics, actin filaments were visualized via immunostaining. Raw 264.7 cells were cultured for 24 h on patterned PDMS surfaces, featuring a Young's modulus of 50 GPa and wrinkles with a characteristic wavelength of 2.5 μm. Actin filaments are depicted in green, while cell nuclei are represented in blue. Quantitative analysis of actin filament orientation revealed that 88% of cells exhibited stretching and alignment along the primary direction of the wrinkles. Moreover, heightened fluorescence intensity observed at the peaks of the wrinkles suggested an accumulation of actin, indicative of focal adhesion sites. These findings underscore the potential for patterned substrates to guide cell movement and may shed light on the mechanisms by which macrophages navigate their microenvironment. The image dimensions are 144.3 × 185.6 *μ*m^2^.

In this study, patterned PDMS surfaces with an elastic modulus of 50 GPa and a characteristic wrinkle wavelength of 2.5 *μ*m were generated by subjecting a 1.5 mm PDMS sample to a 20% strain, followed by a 2-h plasma oxidation. A murine macrophage-like immortalized cell line (Raw 264.7) was cultured on these substrates in Dulbecco's modified Eagle medium (DMEM, Sigma-Aldrich, St. Louis, MO, USA) supplemented with 10% of fetal bovine serum (FBS, Sigma-Aldrich, St. Louis, MO, USA), 1% penicillin-streptomycin (Pen-Strep 10 000 U/ml, Lonza, Basel, Switzerland), 1% ultraglutamine (200 mM, Lonza, Basel, Switzerland), and 1% sodium pyruvate (100 mM, Lonza, Basel, Switzerland). To mitigate potential issues related to changes in functional stability and phenotype associated with immortalized cells, experiments were conducted with cells between passages 12 and 20.^7^

After 24 h of culture, Raw 264.7 cells were fixed with a solution of 4% paraformaldehyde (PFA) in phosphate-buffered saline (PBS) for 5 min at room temperature. Subsequently, PFA was removed, and samples were washed with PBS before being permeabilized with 0.5% PBS/Triton X-100 for 5 min on ice. Actin filaments were stained by incubating cells with Phalloidin (Alexa Fluor™ 488, Invitrogen, A12379) for 1 h at room temperature. Nuclei were then stained with 4′,6-Diamidino-2-Phenylindole, Dihydrochloride (DAPI, Invitrogen, D1306) for 5 min at room temperature. Images (Fig. 1) were acquired using a temperature and gas-controlled fluorescent microscopy system (THUNDER Imaging System, Leica). Image analysis was conducted using ImageJ. Actin directionality was determined by performing fast Fourier transform (FFT) analysis and averaging the angular frequency of oriented cells relative to the orientation of the wrinkled pattern.

## Data Availability

The data that support the findings of this study are available from the corresponding author upon reasonable request.
